# Sodium selenite inhibits proliferation and metastasis through ROS‐mediated NF‐κB signaling in renal cell carcinoma

**DOI:** 10.1186/s12885-022-09965-8

**Published:** 2022-08-09

**Authors:** Xiao Liu, Meng Jiang, Chenggang Pang, Jianning Wang, Lijuan Hu

**Affiliations:** 1 Department of Urology, Shandong Provincial Qianfoshan Hospital, Shandong University, Jinan, 250014 Shandong China; 2 Department of Urology, Zoucheng People’s Hospital, Zoucheng, 273500 Shandong China; 3 Department of Orthopaedics, Zoucheng People’s Hospital, Zoucheng, 273500 Shandong China; 4 Tianjin Key Laboratory of Acute Abdomen Disease Associated Organ Injury and ITCWM Repair, Institute of Acute Abdominal Diseases of Integrated Traditional Chinese and Western Medicine, Tianjin Nankai Hospital, Nankai Clinical College, Tianjin Medical University, Tianjin, China

**Keywords:** Renal Cancer, Sodium selenite, Proliferation, Migration, NF-κB

## Abstract

**Background:**

Sodium selenite (SSE) has been reported to exert anti-tumor effects in several cancer cells. However, the underlying mechanisms in renal cancer are yet to be elucidated. The effects of SSE on the proliferation, metastasis, and apoptosis of renal cancer cells, as well as its mechanism, were investigated in this study.

**Methods:**

ACHN and 786-O renal cancer cells were treated with different concentrations of SSE, MTT, and colony formation assays were used to detect the proliferation ability of cells. The migration of cells was detected using scratch-wound-healing and transwell-migration assays. The effect of SSE on apoptosis was assessed by AnnexinV-FITC/PI double staining. Besides, Western blotting was employed to detect the protein-expression level and elucidate the underlying pathways. We also made subcutaneous xenografts in athymic mice to verify the effect of SSE on tumor growth in vivo.

**Results:**

Our results demonstrated that treatment with SSE resulted in significant inhibition of cell proliferation and migration. Flow cytometry and Western blot confirmed that SSE induced apoptosis via the endogenous apoptotic pathway. We also confirmed that SSE treatment causes an increase in intracellular reactive oxygen species (ROS) levels, resulting in the inhibition of nuclear transcription factor-κB (NF-κB) signaling. Modulation of the ROS level by the chemical inhibitor N-acetyl-cysteine reversed the effect of SSE on cells. Similarly, subcutaneous xenografts in athymic mice models showed that SSE inhibits tumor growth in vivo.

**Conclusion:**

These results indicate that SSE inhibits proliferation and migration and induces apoptosis via ROS mediated inhibition of NF-κB signaling in renal cancer cells.

**Supplementary Information:**

The online version contains supplementary material available at 10.1186/s12885-022-09965-8.

## Background

Renal cell carcinoma (RCC) is one of the most common urinary malignancies and its incidence and mortality are increasing in China [1]. Clear cell renal cell carcinoma is the most common type, accounting for about 80% of RCC cases [2]. With the continuous improvement of tumor detection due to the progress of imaging, more and more patients with early renal cancer have been found and received effective treatment [3]. However, 20–40% of patients still have metastasis at the time of diagnosis, namely metastatic RCC [4]. Surgical resection is the best treatment for localized RCC, but 20–40% of patients still have recurrence and metastasis after surgery [5]. These patients have a poor prognosis, the 5-year survival rate is less than 10% and the median survival time of them is only 1.5 years [6, 7]. RCC is not sensitive to radiotherapy and chemotherapy, which undoubtedly increases the difficulty of treatment. Currently, sorafenib, sunitinib, Tyrosine kinase inhibitors and other targeted therapies have been developed, but their improvement in survival rate is still very limited and they have obvious side effects [8, 9]. Therefore, it is necessary to understand the underlying molecular mechanisms of RCC and identify new therapeutic strategies. More effect on suppressing cancer cells within minimal toxicity to normal cells and tissues is the ultimate goal.

Selenium, as a necessary element for human body, is of great significance benefit to human health [10]. Clinical studies have shown that low-dose selenium intake is negatively correlated with the risk of cancer [11, 12]. Sodium selenite (SSE), a source of inorganic selenium, has been widely used as a clinical cancer treatment. Many previous studies have confirmed that SSE induced apoptosis in some types of cancer cells through different mechanisms [13]. For example, SSE induced apoptosis and autophagy in lung cancer A549 cells through increasing intracellular reactive oxygen species (ROS) levels [14]. The inhibition of SSE on colon cancer HCT116 and SW620 cells is related to the activation of C-Jun NH 2-terminal kinase [15]. However, the effect of SSE on RCC has not been reported. Some researchers have found that the concentration of selenium in RCC is lower than that of healthy kidney tissues, which indicates that selenium supplementation may have a certain effect on the treatment of RCC [16].

The present study aimed to investigate the anticancer effect of SSE in 786-O and ACHN cell lines both in vitro and in vivo. We assessed the anti-proliferation, anti-migration and apoptosis-induction abilities of SSE in 786-O and ACHN cells in vitro, and then the involvement of ROS-mediated NF-κB signaling was evaluated after SSE treatment. Besides, the anti-tumor activities of SSE in athymic mice bearing 786-O cells were evaluated. We hope that this study can provide some theoretical support for the adjuvant therapy of selenium.

## Methods

### Reagents and antibodies

Na_2_SeO_3_ was produced by Sigma-Aldrich (MKCJ9649, St Louis, MO, USA). Mitochondrial membrane potential assay kit with JC-1 (C2006), Annexin V-FITC apoptosis detection kit (C1062), N-acetyl-cysteine (NAC, #ST1546), and reactive oxygen species (ROS) assay kit (#S0033) were purchased from Beyotime (Shanghai, China). The manufacturers and numbers of the antisera are given in the following parentheses, with each parenthesis being preceded with the name of the antigen: phosphorylated (p-) NF-κB p65 (Affinity Biosciences, AF2006), NF-κB p65 (Affinity Biosciences, AF5006), phosphorylated (p-) IκB-α (Affinity Biosciences, AF2002), IκB-α (Affinity Biosciences, AF5002), MMP-9 (Affinity Biosciences, AF5228), E-cadherin (Affinity Biosciences, AF0131), Bcl-2 (Affinity Biosciences, AF6139), VEGF (BD Pharmingen, 555036), Bax (CST, 92772), cleaved-caspase3 (CST, 9662S), β-tubulin (Protein tech, 66240–1) and β-actin (Abcam, 8227). Dulbecco modified Eagle's media (DMEM), RPMI-1640 media, Fetal bovine serum (FBS), penicillin, and streptomycin were bought from Gibco Thermo Fisher Scientific (Beijing, China). Transwell chambers were obtained from Corning Inc. (Corning, NY, USA). BAY 11–7082 (MCE, #HY-13453).

### Cell culture and treatment

The human renal cancer cell lines 786-O and ACHN were purchased from Procell Life Science Technology (CL-0010 and CL-0021, Wuhan, China). 786-O cells and ACHN cells were cultured in RPMI-1640 medium and high glocose DMEM medium respectively, containing 10% (v/v) FBS and 1% penicillin–streptomycin at 37 °C in 5% CO_2_ humidified incubator.

### Cell viability assay

786-O or ACHN cells were seeded in 96-well culture plates at 5 × 10^3^ cells/well with medium containing 10% FBS. After 24 h incubation, cells were treated with different concentrations of SSE for 6 h, 12 h and 24 h. Following incubation, MTT (5 mg/ml) solution was added to each well and the plate was incubated at 37 °C for another 2 h. At last, DMSO was added to each well and the absorbance at 570 nm was measured using an enzyme-labeled instrument.

### Clone formation assay

786-O cells were seeded in 6-well culture plates at 250 cells/well and ACHN cells were seeded in 6-well culture plates at 450 cells/well. All cells were incubated for 24 h at 37 °C in 5% CO_2_ humidified incubator. Different concentrations of SSE were added to each well. Following SSE treatment for 24 h, the cell medium was removed and the cells were cultured with new medium for 2 weeks at 37 °C in 5% CO_2_ humidified incubator. Cells were fixed in 4% paraformaldehyde for 20 min and then stained with 0.1% crystal violet for 30 min at room temperature. Colonies were counted: those containing ≥ 25 cells were classed as a colony.

### Scratch wound healing assay

786-O or ACHN cells were seeded in 6-well culture plates at 2 × 10^5^ cells/well and grown to 90% confluence in medium containing 10% FBS. After being starved in serum-free medium overnight, cells were scraped using a sterile pipette tip to produce a clean-wound area across the center of the well, then the original medium was replaced with different concentration of SSE for 24 h, wound healing was observed and photographed under a microscope. The results were analysed using ImageJ software.

### Transwell assay

Cell migration assays were performed using 24-well cell culture plate with transwell chambers. Chambers were precoated with or without matrigel (Matrigel was necessary for the transwell-invasion assay). 786-O or ACHN cell suspension (200 μl) containing 2 × 10^4^ cells with different concentration of SSE was placed in the upper chamber and 600 μl DMEM or 1640 medium supplemented with 20% FBS was added to the lower chamber. After incubated for 24 h at 37 °C, cells on the upper surface of chambers were wiped away gently, whereas cells that migrated to the lower surface of chambers were fixed with 4% paraformaldehyde for 20 min and stained with 0.1% crystal violet for 30 min at room temperature. Images of the migrated cells were captured using a microscope.

### Annexin V-FITC/PI double staining

786-O cells or ACHN cells were seeded in 6-well culture plates and grown to 90% confluence, cells were treated with different concentrations of SSE for 6 h. After that cells were collected and washed with cold phosphate buffer saline (PBS) and then stained in 210 μl solution containing Annexin V-FITC and PI for 15 min in the dark at room temperature according to the manufacturer’s instructions. Subsequently, the fluorescent signal was detected by flow cytometry (Becton Dickinson; Franklin Lakes, NJ, USA).

### Detection of intracellular ROS level

786-O cells or ACHN cells were seeded in 6-well culture plates and grown to 90% confluence, cells were treated with different concentrations of SSE for 6 h. The intracellular ROS levels were measured using the oxidative conversion of cell-permeable 2′, 7′-dichlorofluorescein diacetate (DCFH-DA) into fluorescent dichlorofluorescein (DCF). After that cells were incubated with the fluorescent probe DCFH-DA for 20 min and then washed three times with PBS to remove the unbound DCFH-DA. The DCF fluorescence was detected using fluorospectro photometer.

### Mitochondrial membrane potential assay

The mitochondrial membrane potential level was measured by fluorospectro photometer according to the JC-1 Kit Manufacturer’s Manual. Briefly, 786-O cells or ACHN cells were treated with different concentrations of SSE for 6 h, cells were incubated with JC-1 for 30 min at 37 °C and then washed twice. The fluorescence was detected using fluorospectro photometer.

### Western blot

Whole-cell proteins were extracted using RIPA lysis buffer with phosphatase inhibitors. Then, equal amounts of sample protein were separated in polyacrylamide gel, then transferred to a piece of polyvinylidene difluoride membrane. The membrane was cut into different strips according to the molecular weight of the target protein, and then hybridized with the corresponding antibody at 4 °C overnight and a secondary antibody for 1 h at room temperature. The specific blotting was visualized using an enhanced ECL detection kit. When the molecular weights of the target proteins were adjacent or the same to each other that the strips could not be cut according to the molecular weight, the strips were washed with stripping buffer to remove the original antibody, and then re-hybridized with the other antibody. Blots were digitalized using the software of ImageJ.

### Real-time RT PCR

Different concentrations of SSE were used to incubate cells for 6 h, after that total RNA was extracted from the collected cells using TRIzol reagent, cDNA was synthesized using a reverse transcription kit (Thermo #K1622). The primer sequences used are in Table 1. The reaction had 40 cycles, with each cycle having 10 s at 95 °C and 20 s at 60 °C. The mRNA of β-actin was used for control.

**Table 1:** Primer sequences used for Real-time RT PCR

genes	Forward sequence (5’ − 3’)	Reverse sequence (5’ − 3’)
Bax	TCAGGATGCGTCCACCAAGAAG	TGTGTCCACGGCGGCAATCATC
Bcl-2	ATCGCCCTGTGGATGACTGAGT	GCCAGGAGAAATCAAACAGAGGC
cIAP	CAGACACATGCAGCTCGAATGAG	CACCTCAAGCCACCATCACAAC
xIAP	TGGCAGATTATGAAGCACGGATC	AGTTAGCCCTCCTCCACAGTGA
E-cad	GCCTCCTGAAAAGAGAGTGGAAG	TGGCAGTGTCTCTCCAAATCCG
MMP-9	GCCACTACTGTGCCTTTGAGTC	CCCTCAGAGAATCGCCAGTACT
VEGF	TTGCCTTGCTGCTCTACCTCCA	GATGGCAGTAGCTGCGCTGATA
β-actin	GATAGCACAGCCTGGATAGCA	ACTGGGACGACATGGAGAAAA

### Immunofluorescence staining

786-O cells or ACHN cells were seeded on glass coverslips in 6-well culture plates. After cells were treated with different concentrations of SSE for 6 h, they were fixed with 4% paraformaldehyde and treated with 0.1% Triton X-100 to permeabilize for 15 min. After blocking with 1% BSA for 1 h, cells were incubated in turn with phospho-NF-κB p65 antibody overnight at 4 °C, IFKine™ green donkey anti-rabbit IgG for 1 h, and DAPI solution for 20 min at room temperature. Then stained phospho-NF-κB p65 and nucleus puncta were detected under a fluorescence microscope and merged.

### Animal study

BALB/c athymic mice (6–8 weeks of age) were purchased from Hua-Fu-Kang Bioscience (Beijing, China). All mice lived in a pathogen-free condition with a 12 h light/dark cycle and had free access to food and water. 786-O cells were suspended in serum-free culture medium (5 × 10^6^ cells in 200 μl) and injected in an athymic mouse subcutaneously. After tumor volume reached to approximately 20 mm^3^, the tumor-bearing mice were randomly assigned into two groups (n = 5): Control group (200 μl of saline intraperitoneal injection every other day for two weeks), SSE group (200 μl of 2 mg/kg SSE intraperitoneal injection every other day for two weeks). Tumor size and the weight of mice were measured every three days. Tumor volume (V) was calculated according to the formula V = 0.5 × length × width^2^. At the end of the treatment, all mice were sacrificed. Tumor grafts were harvested and weighed. Each tumor was cut, half tumor was put in 10% paraformaldehyde for histology, and the other half saved at -80℃ for Western blot.

### Histology

The level of tumor tissue apoptosis was determined using a kit of TUNEL (S7100, Millipore, Temecula, CA) and counter-stained with hematoxylin according to the Manufacturer’s Manual. Tumor morphology was captured using a microscope (DFC500; Leica Camera).

### Statistical analysis

Each experiment was performed three independent times. The results were analyzed using SPSS 23.0 and GraphPad Prism 9. Comparisons among all groups were performed with one-way ANOVA or T test. The data were expressed as the means ± SD. *p* < 0.05 was considered statistically significant.

## Results

### SSE inhibited renal cancer 786-O and ACHN cell proliferation

**Fig.1:**
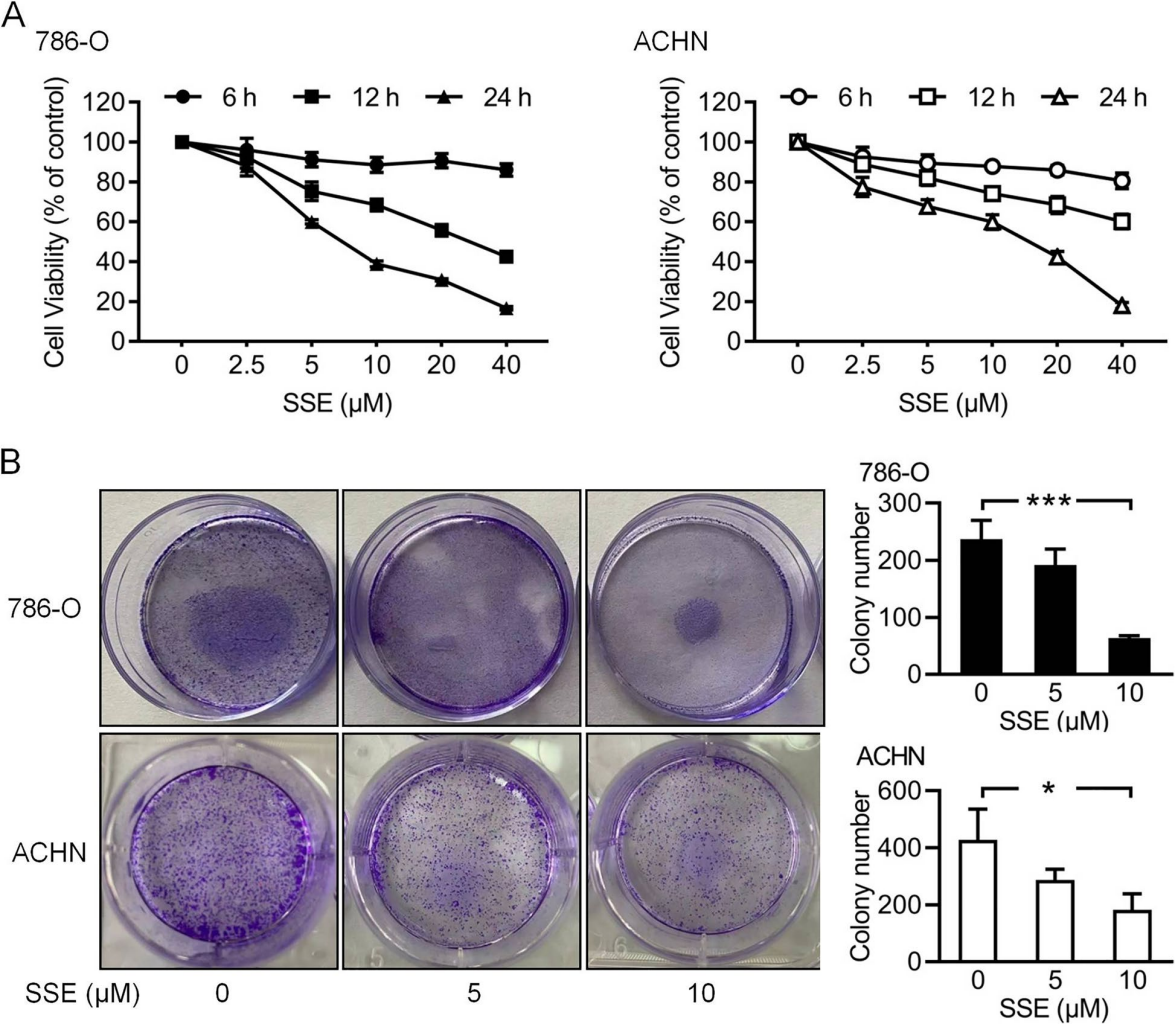
SSE decreased the viability and proliferation of two renal cancer cell lines. 786-O and ACHN cells were treated with a range of SSE doses for up to 24 h. **A** Cell viability was determined using an MTT assay. Data from cells without SSE were as a baseline (100%). **B** The inhibitory effects of SSE on cell proliferation were determined using a colony formation assay. The black bars and white bars denote the quantitative analysis of 786-O cells and ACHN cells respectively. Data were expressed as means ± SD (*n* = 4), *P < 0.05 and ***P < 0.001

To investigate the effect of SSE on renal cancer, we first evaluated the cell viabilities by treating 786-O and ACHN cell lines in 6 h, 12 h and 24 h with different concentrations of SSE. As shown in Fig. 1A, the viability of the two cell lines treated with SSE were significantly reduced in a time- and dose-dependent manner compared to the cells without SSE. The IC50 of SSE was 5.15 μM in 786-O cells and 17.27 μM ACHN cell in 24 h. The SSE dose-dependent inhibition was also demonstrated by the colony formation assays (Figs. 1B). Notably, the proliferation ability of the two cell lines was significantly decreased with SSE. Taken together, these data showed that SSE inhibited 786-O and ACHN cell proliferation.

### SSE inhibited renal cancer 786-O and ACHN cell migration and invasion

**Fig. 2:**
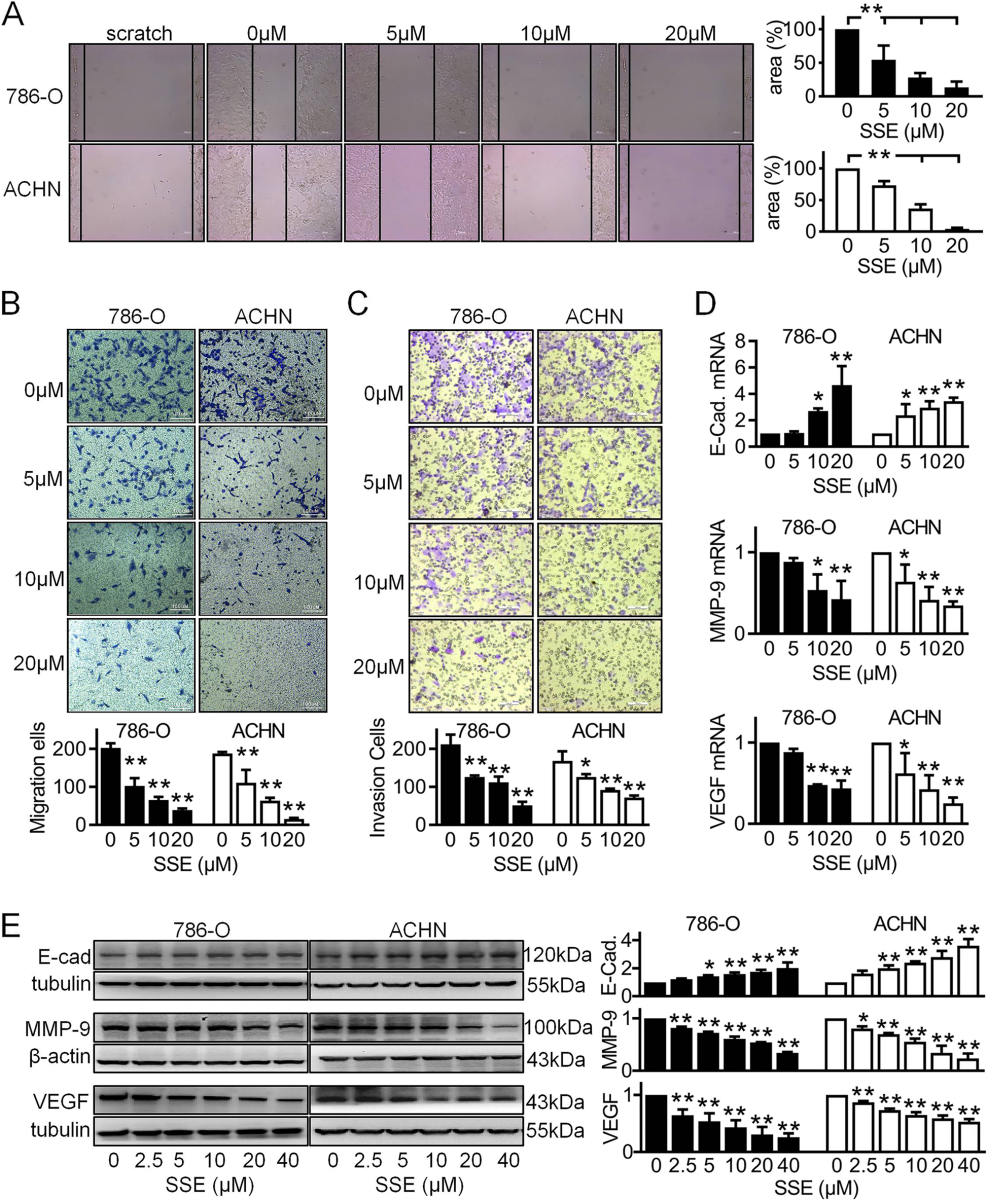
SSE repressed the migration and invasion of renal cancer cells. **A** A wound healing assay, a transwell assay without matrigel for cell migration (**B**) and a transwell assay with matrigel for cell invasion (**C**) were performed on 786-O and ACHN cells following SSE treatment for 24 h. The images shown are representatives (Scale bar = 100 μm). Following SSE treatment for 6 h, E-cadherin, MMP-9 and VEGF were determined in cells using real-time RT PCR (**D**) and Western blot (**E**). β-Actin mRNA and β-actin were used as loading controls. The blots shown are representatives. The black bars and white bars denote 786-O cells and ACHN cells respectively. Data are presented as means ± SD (*n* ≥ 3). *P < 0.05 and **P < 0.01 vs the control group

To determine the efficacy of SSE against cancer-cell metastasis in vitro, the wound-healing and transwell-migration assays were introduced. As shown in Fig. 2A, SSE inhibited 786-O-cell migration since 5 μM after 24 h incubation, the inhibition was enlarged when the SSE concentration was increased. SSE also inhibited ACHN-cell migration in a dose-dependent manner (Fig. 2B). Also, the results from the transwell-migration assays were in line with the data from the scratch assay. In Fig. 2C, SSE inhibited the two cell lines invasion efficiently with the increased SSE concentration. Epithelial mesenchymal transition (EMT) as well as angiogenesis is generally considered to be related to tumor cell invasion and migration. Here, we used Real-time RT PCR and Western blot to assess the expression of EMT related protein MMP-9 and E-cadherin and angiogenesis related protein VEGF. As shown in Fig. 2D, we found that the mRNA of E-cadherin was increased while as MMP-9 and VEGF were decreased with the increased SSE concentration. Also, the expressions of E-cadherin, MMP-9 and VEGF in Western blot (Fig. 2E) were coincidence with Real-time RT PCR. These data showed that SSE inhibited 786-O and ACHN cell migration and invasion.

### SSE induced apoptosis in 786-O and ACHN cell

**Fig. 3:**
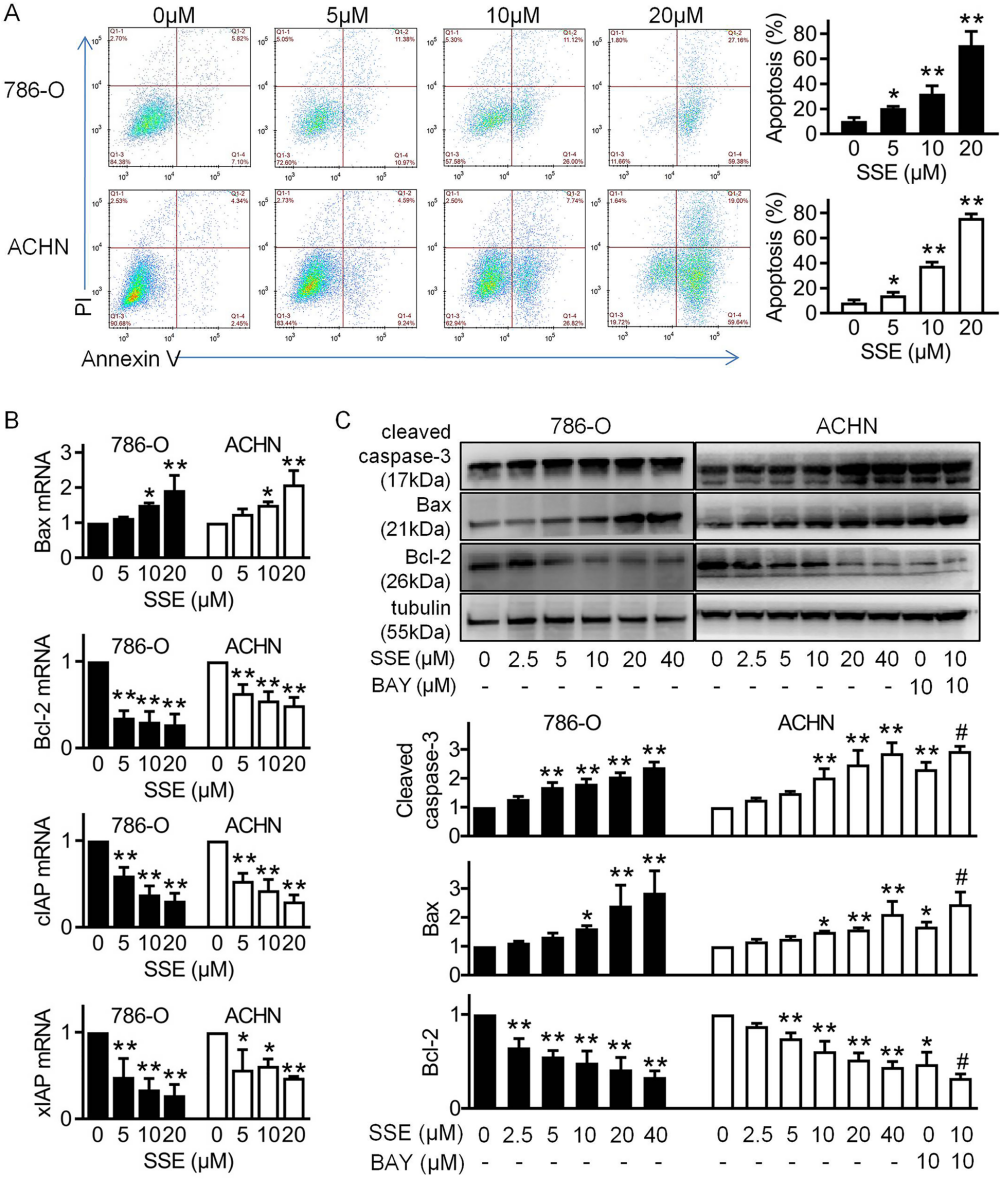
Induction of apoptosis on 786-O and ACHN cells by SSE. **A** Flow cytometry images and quantitative analysis of the percentage of apoptotic cells of SSE on 786-O (black bars) and ACHN (white bars) cells after 6 h incubation. The percentage of total apoptotic cells was defined as the sum of early and late apoptotic cells. The expressions of apoptosis related protein were determined in cells using real-time RT PCR (**B**) and Western blot (**C**). In ACHN cells, BAY (10 μM) was used without or with SSE (10 μM) for 6 h. β-Actin mRNA and β-tubulin were used as loading controls. The blots shown are representatives. The black bars and white bars denote 786-O cells and ACHN cells respectively. Data are presented as means ± SD (*n* ≥ 3). *P < 0.05 and **P < 0.01 vs the control group. # *P* < 0.05 vs 10 μM SSE alone group

Previous studies have shown that SSE induced cells apoptosis in other cancers, to verify the induction of SSE in RCC, Annexin V- FITC/PI staining was performed. The results showed that when 786-O and ACHN cells were incubated with an increasing dose of SSE, the rates of cell apoptosis were increased in a dose-dependent manner (Fig. 3A). We also used Real-time RT PCR and Western blot to assess the expression of apoptosis related protein. As shown in Fig. 3B, we found that the mRNA of bax was increased while as bcl-2, cIAP and xIAP were decreased with the increased SSE concentration. Also, SSE treatments increased the expressions of cleaved caspase-3 and bax and decreased bcl-2 in Western blot, these results are consistent with the NF-κB inhibitor BAY-11–7082 (BAY) group (Fig. 3C). These results suggest that SSE induces cell apoptosis by inhibiting NF-κB signaling.

### SSE induced apoptosis and inhibited migration by increasing ROS levels

**Fig. 4:**
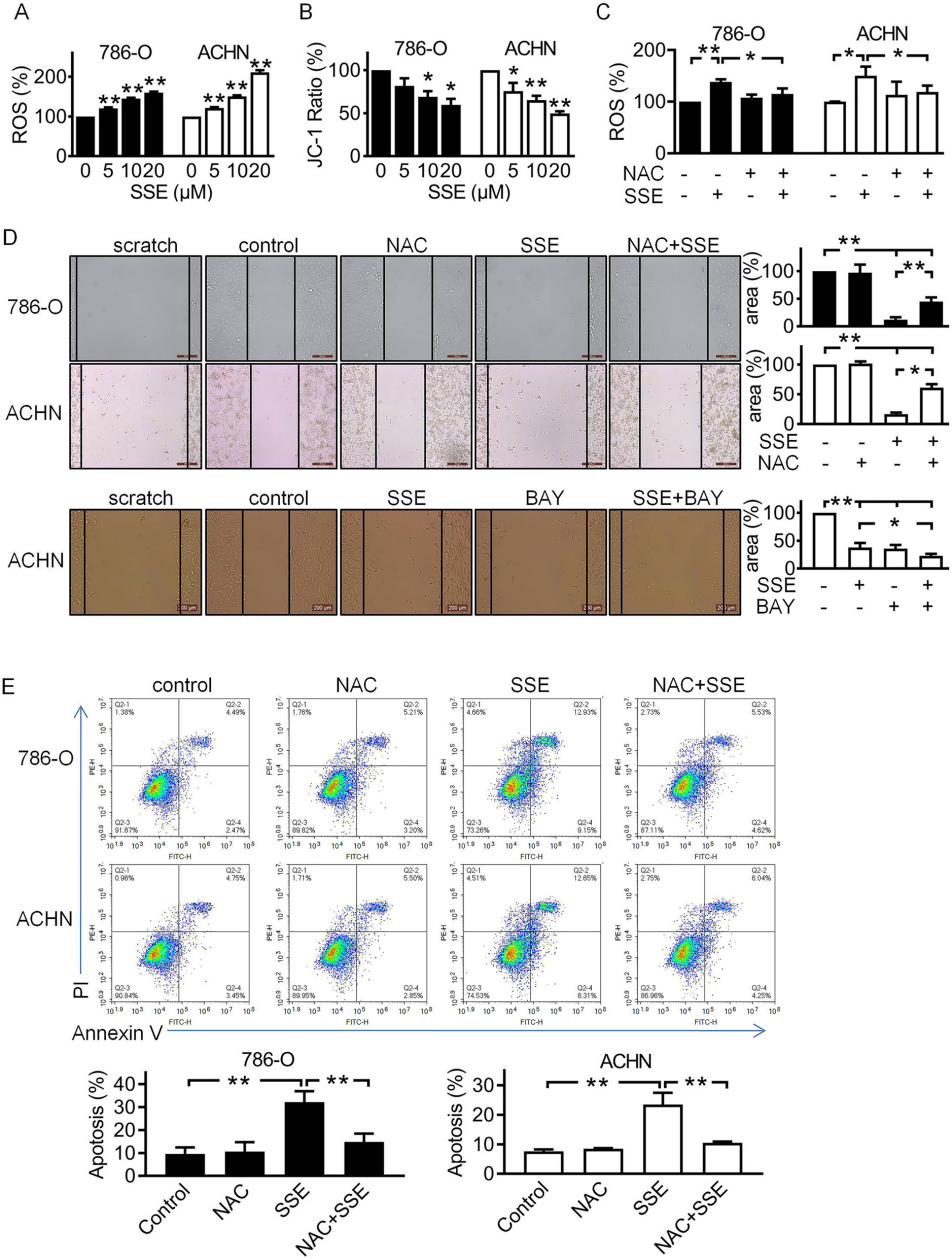
SSE induced apoptosis and inhibited migration by increasing ROS levels. Following cells were incubated with different concentration of SSE for 6 h, the intracellular ROS levels (**A**) and mitochondrial membrane potential (**B**) were determined by fluorospectro photometer. **C**-**E** The cells were treated with 10 μM NAC, 10 μM SSE, or 10 μM NAC and 10 μM SSE for 6 h, the intracellular ROS levels were determined by fluorospectro photometer (**C**), cell migration was determined by wound healing assay (**D**), and cell apoptosis was determined by flow cytometry (**E**). The black bars and white bars denote 786-O cells and ACHN cells respectively. Data are presented as means ± SD (*n* ≥ 3). *P < 0.05 and **P < 0.01

Previous studies indicate that ROS plays a crucial role in apoptosis and migration pathways. Thus, we hypothesized that SSE-induced apoptosis and inhibited migration are associated with high levels of intracellular ROS. We detected intracellular ROS and mitochondrial membrane potential (MMP). As shown in Figs. 4A and 4B, SSE increased the intracellular ROS and reduced MMP in both cells. However, NAC, a ROS scavenger, could reverse SSE induced ROS levels (Figs. 4C). In addition, SSE induced apoptosis and inhibited migration could be reversed by NAC (Figs. 4D and 4E). In addition, to confirm the role of ROS in the effect of SSE on cell migration and apoptosis, we also use NAC to evaluate the protein expression of migration- and apoptosis-related molecules. As shown in supplementary Figures A and B (s. Figs. A and B), inhibition of ROS levels with NAC reversed the effect of SSE on the protein expression of migration- and apoptosis-related molecules. We also detected the ACHN cells migration using BAY without or with SSE, the SSE combination with BAY significantly inhibited migration compared with the other groups (Fig. 4D). These results indicated that SSE induced apoptosis and inhibited migration by increasing ROS levels.

### SSE inhibited ROS-mediated NF-κB signaling pathway

**Fig. 5:**
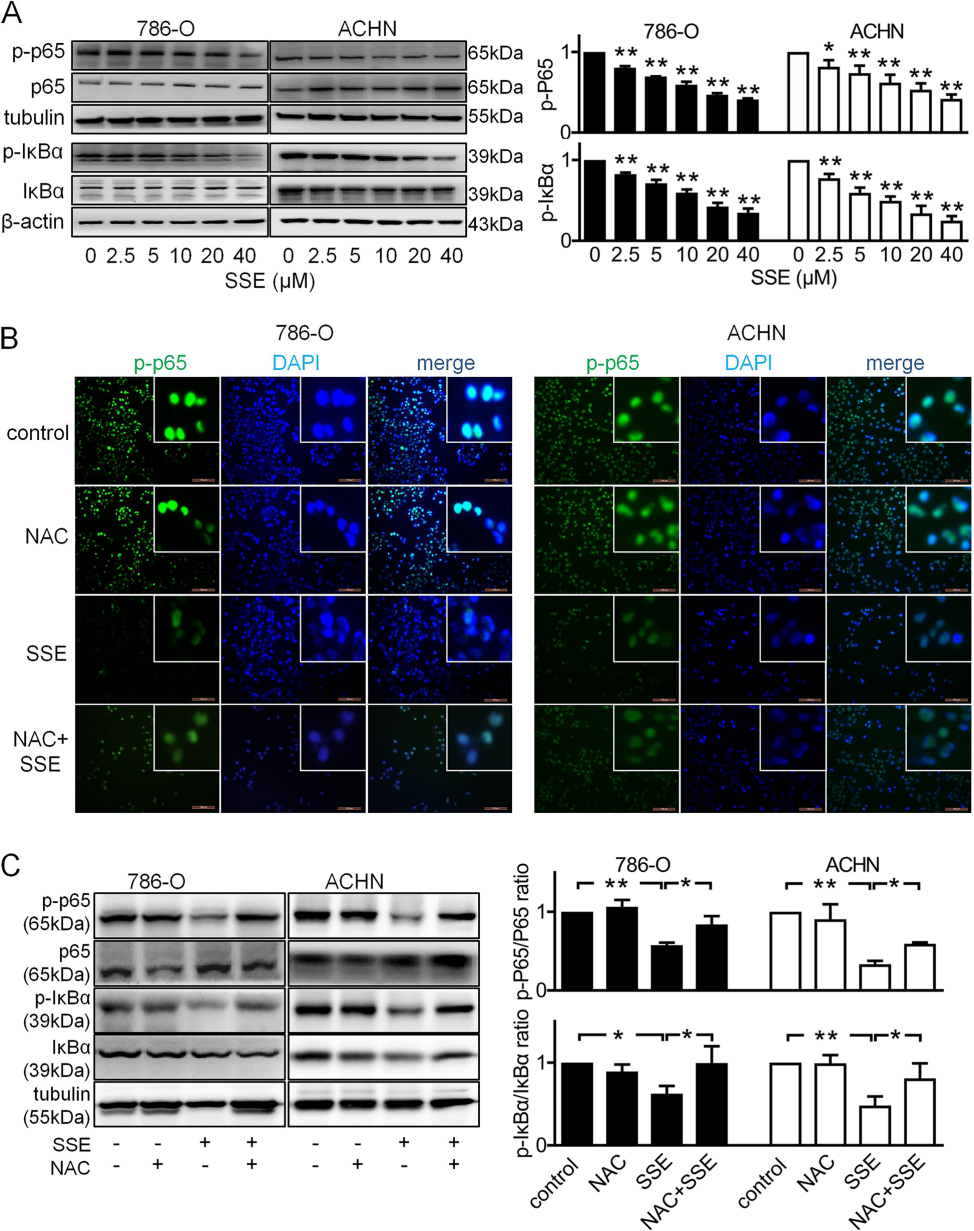
The effect of SSE on activation of the NF-κB pathway. **A** The cells were treated with different concentrations of SSE for 6 h, the protein expression levels of p65 and IκBα were analyzed using specific antibodies by western blot. **B**-**C** The cells were treated with 10 μM NAC, 10 μM SSE, or 10 μM NAC and 10 μM SSE for 6 h, the p65 subunit translocation from the cytoplasm to the nucleus was evaluated by immunofluorescence, green spots represent p-p65 staining and blue spots represent the cell nuclei (**B**). The images shown are representatives (Scale bar = 100 μm). The protein expression levels of p65 and IκBα were analyzed using specific antibodies by western blot (**C**). The black bars and white bars denote 786-O cells and ACHN cells respectively. Data are presented as means ± SD (n ≥ 3). *P < 0.05 and **P < 0.01

Previous studies have shown that high levels of intracellular ROS can repress the NF-κB signaling pathway [17]. NF-κB is a master regulator of many proteins involved in apoptosis and metastasis. To elucidate whether the underlying molecular mechanisms of SSE on RCC are dependent on the activation of NF-κB, the levels of phosphorylated p65 and IκB-α were detected using Western blot. As shown in Fig. 5A, SSE reduced the levels of p-p65 and p-IκB-α in a dose-dependent manner.

Next, we explored whether the suppression of NF-κB signaling was caused by high levels of intracellular ROS. Immunofluorescence in shown Fig. 5B demonstrated that SSE inhibited translocation of p-p65/NF-κB from the cytoplasm into the nucleus. These results were consistent with the BAY group, in addition, the SSE combination with BAY significantly inhibited translocation of p-p65/NF-κB (s. Fig. C). Inhibition of ROS levels with NAC also suppressed the nuclear translocation of p-p65/NF-κB even with SSE (Fig. 5B). Western blot shown in Fig. 5C demonstrated that the levels of p-p65 and p-IκB-α in the SSE + NAC group were also upregulated compared with that of the SSE-alone. These results indicated that SSE increased intracellular ROS levels and then inhibited the NF-κB signaling pathway in both cells.

### The inhibitory effect of SSE in vivo

**Fig. 6:**
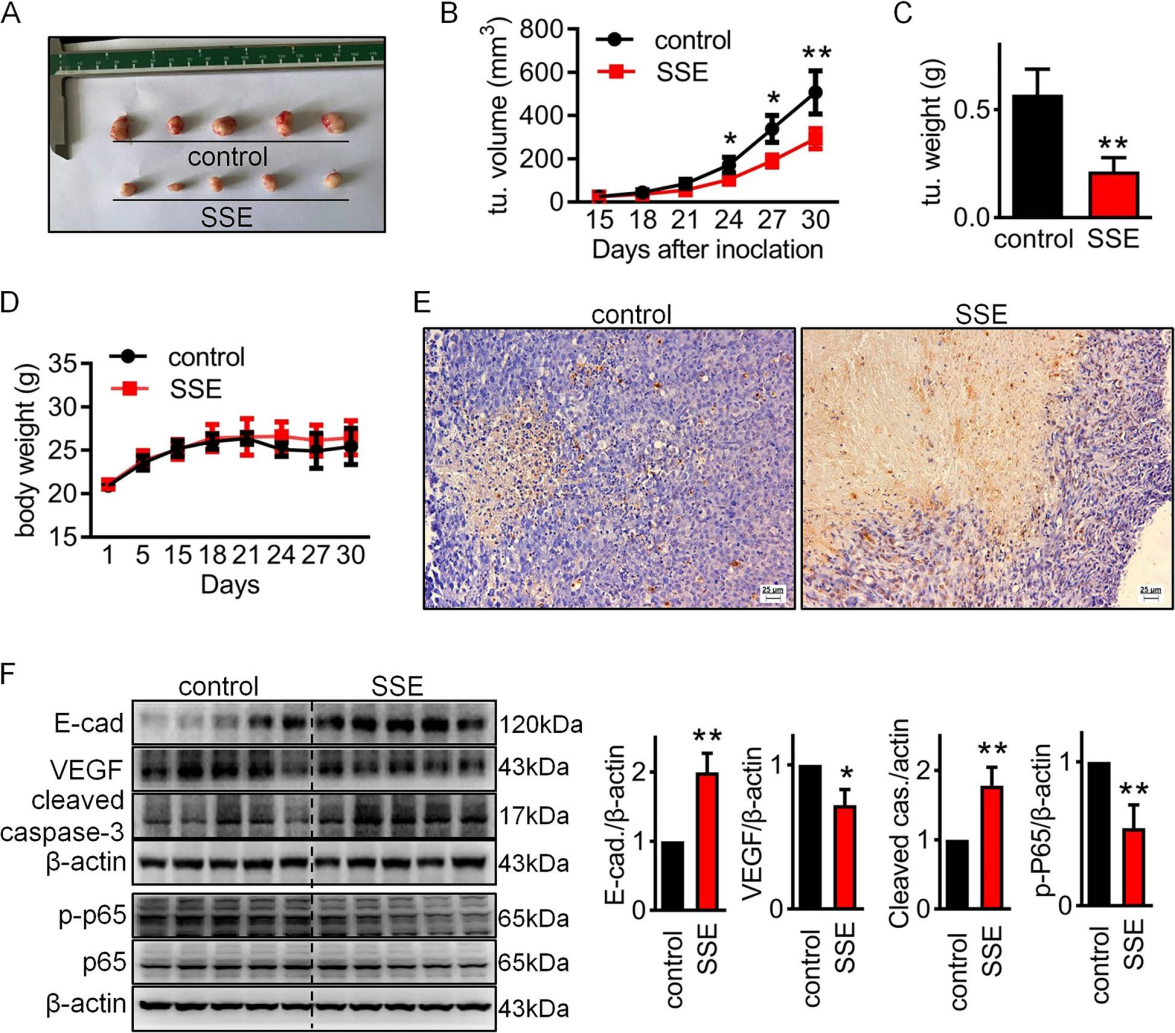
The inhibitory effect of SSE in vivo. 786-O cells grew as subcutaneous tumors in two groups of athymic mice (5 mice per group) for 30 days. In the last 2 weeks, the mice were treated with saline or 2 mg/kg SSE every other day. **A** Tumor images in the two groups. Tumor volumes (**B**), tumor weights (**C**) and mouse weights (**D**) were measured in the two groups. **E** Representative images (Scale bar = 100 μm) of the tumor stained with TUNEL. Blue spots represent cell nuclei and yellow spots represent TUNEL-positive cells. **F** The expression of E-cadherin, VEGF and cleaved caspase-3, p-p65 and p65 were analyzed by western blot. β-Actin was used as loading control. Data are presented as means ± SD. *P < 0.05 and **P < 0.01.

To investigate the anticancer effect of SSE in vivo, 786-O cells were injected into the right flank of BALB/c athymic mice. As shown in Figs. 6A and 6B, xenograft tumors treated with SSE exhibited slower growth than tumors in the control group. The final tumor weight was obviously decreased in SSE group (Figs. 6C). There were no significant differences in body weight between SSE and control group (Figs. 6D). Next, we evaluated the effect of SSE on cell apoptosis in the xenograft tumors using TUNEL assay. The results showed that the percentage of TUNEL positive cells was significantly higher in SSE treated xenografts (Figs. 6E). We also used western blot to test the expression of E-cadherin, VEGF and cleaved caspase-3. As shown in Fig. 6F, SSE treatment significantly increased the expression of E-cadherin and cleaved caspase-3, decreased the expression of VEGF. These results were in line with the findings in vitro. Taken together, these data showed that SSE treatment inhibited RCC in vivo.

## Discussion

Renal cell carcinoma (RCC) is one of the most common urinary malignancies with high morbidity and mortality and poor prognosis [18, 19]. RCC is not sensitive to radiotherapy and chemotherapy, which undoubtedly increases the difficulty of treatment. Clinical studies have shown that low-dose selenium intake is negatively correlated with the risk of cancer [11, 12]. We hypothesized that selenium supplementation may improve the therapeutic effect of RCC. In order to confirm this hypothesis, we applied sodium selenite (SSE) which is a source of inorganic selenium, to renal cancer cells, and found that SSE not only inhibited the proliferation, migration and invasion but also induced apoptosis in renal cancer cells. This effect of SSE on renal cancer was through ROS mediated NF-κB signaling pathway.

In this study, we first performed MTT assay and colony formation assay to find that SSE inhibits the renal cancer cells in a time-dependent and dose-dependent manner. This is similar to previous studies on other cancers [14]. We also used Transwell assay and wound healing assay to find that SSE inhibited the metastasis ability of renal cancer cells. Migration and invasion are two major events in tumor metastasis [20]. Epithelial-mesenchymal transition (EMT) has been found to participate in metastasis in malignant tumor cells [21]. E-cadherin is one of the indirect markers of EMT, once the amount of E-cadherin was reduced, intercellular adhesion was decreasing, tumor cells become more invasive [22]. In addition, many members of the matrix metalloproteinases family are associated with metastasis of cancer (e.g., MMP-2, MMP-9) [23, 24]. In this study, we found that SSE increased the expression of E-cadherin while as decreased the expression of MMP-9, suggesting that SSE may inhibit the metastasis of RCC by regulating EMT and extracellular matrix degradation. In addition, MMPs play an important role in the formation of tumor blood vessels [25]. During tumor development pro-angiogenic factors such as vascular endothelial growth factor (VEGF), a key angiogenic factor, is pathologically enhanced. Persistent growth of tumor directed capillary networks creates a favorable microenvironment, promoting cancer growth, progression and metastasis. Targeting VEGF is a viable strategy to prevent tumor growth and metastasis [26]. In this study, we found that SSE decreased the expression of VEGF, suggesting that SSE may inhibit VEGF and its downstream signaling cascade to prevent tumor growth and metastasis.

We found that SSE induced apoptosis of renal carcinoma cells in a dose-dependent manner. It is consistent with previous studies [14]. Some studies have reported that SSE can induce apoptosis through the extrinsic pathway by increasing the expression of the death receptors, such as Fas, DR5, and DR4, then activating the caspase family proteins [27]. In this study we found that SSE decreased mitochondrial membrane potential and increased the intracellular ROS, which suggested that SSE induced mitochondrial damage. Mitochondrial damage is associated with the intrinsic pathway [28]. The intrinsic pathway is regulated by the activities of bcl-2 family including pro-apoptotic proteins Bax, Bak, Bad, Bid, etc., and anti-apoptotic proteins bcl-2, Bcl-XL, etc. [29]. In addition, inhibitors of apoptosis proteins (IAPs) are endogenous inhibitors of Caspase which bind to the active site of Caspase thereby inhibiting its activity [30]. Therefore, we detected the pro-apoptotic proteins Bax, Caspase-3, and found that SSE increased their expression. At the same time, we detected anti-apoptotic proteins Bcl-2, and found that SSE decreased its expression. These result suggested that SSE induced apoptosis of renal cancer cells through intrinsic apoptotic pathway. This is similar to previous studies on other cancers [17, 31] Caspase-9 is a well-known initiator Caspase which triggers intrinsic apoptosis. In the endogenous apoptosis pathway, over-expression of Bax can antagonize the protective effect of Bcl-2, then cause mitochondria dysfunction and promote the release of cytochrome C. Subsequently, cytochrome C induces the cleavage of Caspase-9 and then activates Caspase-3 to cause cell apoptosis [32]. Autophagy and apoptosis are two key cellular processes for cell survival and death [33]. Recent studies also suggest various non-apoptotic roles of Caspase-9, including macroautophagy/autophagy regulation [34]. Both autophagy and apoptosis have a role in tumor suppression, as autophagy helps in eliminating the tumor cells, and apoptosis prevents their survival. However, the molecular mechanism of SSE regulating autophagy through Caspase-9 remains unclear. We will be the focus on this in the future.

NF-κB is a master regular regulating multiple intracellular signaling pathways in cells [35]. Abnormal NF-κB signaling is involved in the regulation of tumor growth, metastasis, apoptosis, and chemotherapy-resistant [36]. NF-κB inhibits mitochondrial permeability transition by inducing the expression of anti-apoptotic genes such as xIAP and Bcl-2 [37]. In addition, it also can promote the expression of MMP-9 and VEGF to promote tumor proliferation and migration [38]. Many studies have confirmed that chemotherapy drugs can inhibit NF-κB pathway [36, 39]. As secondary messengers, ROS is involved in the NF-κB pathway and plays a crucial role in apoptosis signaling pathways in many cancers [40]. The mechanism of SSE induced apoptosis in RCC is unclear. Therefore, we hypothesized that SSE induced apoptosis and decreased metastasis are dependent on ROS mediated NF-κB signaling pathway. We confirmed that SSE increased intracellular ROS and decreased NF-κB P65 and IκB-α phosphorylation. We inhibited SSE induced ROS by the ROS scavenger NAC and observed that the SSE induced changes in the NF-κB pathway were reversed and that the SSE induced apoptosis and inhibited migration were reversed too.

It has been reported that neoplastic tissues contain a high amount of selenium compared to their surrounding non-neoplastic tissues. By selenium supplementation, the viability of cancer cells was inhibited in vitro in a dose-dependent manner [31, 41]. However, a normal cell line was relatively resistant to selenium concentration, which indicated that malignant cells are more sensitive to selenium than normal cells [42]. In our vivo study, tumor-bearing mice were treated with 2 mg/kg SSE, this dose is safe for normal organs as we examined the sections of lung tissue by hematoxylin–eosin staining (data were not shown). We also found that SSE inhibited migration and induced apoptosis through inhibiting NF-κB signaling pathway, which is consistent with the results in vitro.

## Conclusion

This study provides evidence that SSE effectively suppresses RCC growth, metastasis and induces apoptosis. We also revealed the mechanism involving increased intracellular ROS, and inhibition of the NF-κB signaling pathway. The results provide a basis for the management of RCC by SSE supplementation.

## Supplementary Information

**Additional file 1. **Supplementary Figure (A) and (B) The ACHN cells were treated with 10 μM SSE, 10 μM NAC, or 10 μM SSE and 10 μM NAC for 6 h. β-Actin or β-tubulin was used as loading controls. The blots shown are representatives. (C) The ACHN cells were treated with 10 μM BAY, or 10 μM BAY and 10 μM SSE for 6 h, the p65 subunit translocation from the cytoplasm to the nucleus was evaluated by immunofluorescence, green spots represent p-p65 staining and blue spots represent the cell nuclei. The images shown are representatives (Scale bar = 100 μm). Data are presented as means ± SD. *P <0.05 and **P < 0.01.

## Data Availability

The datasets used and/or analyzed during the current study are available from the corresponding author on reasonable request.

## Abbreviations

MMP-9: Matrix metalloproteinase 9
NAC: N-acetyl-cysteine
NF-κB: Nuclear transcription factor-κB
RCC: Renal cell carcinoma
ROS: Reactive oxygen species
SSE: Sodium selenite
VEGF: Vascular endothelial growth factor
