# Supplementary figures and images for: Sodium selenite inhibits proliferation and metastasis through ROS‐mediated NF‐κB signaling in renal cell carcinoma
Source: BMC Cancer. 2022 Aug 9;22:870. doi: 10.1186/s12885-022-09965-8 (PMC9364612; doi:10.1186/s12885-022-09965-8)

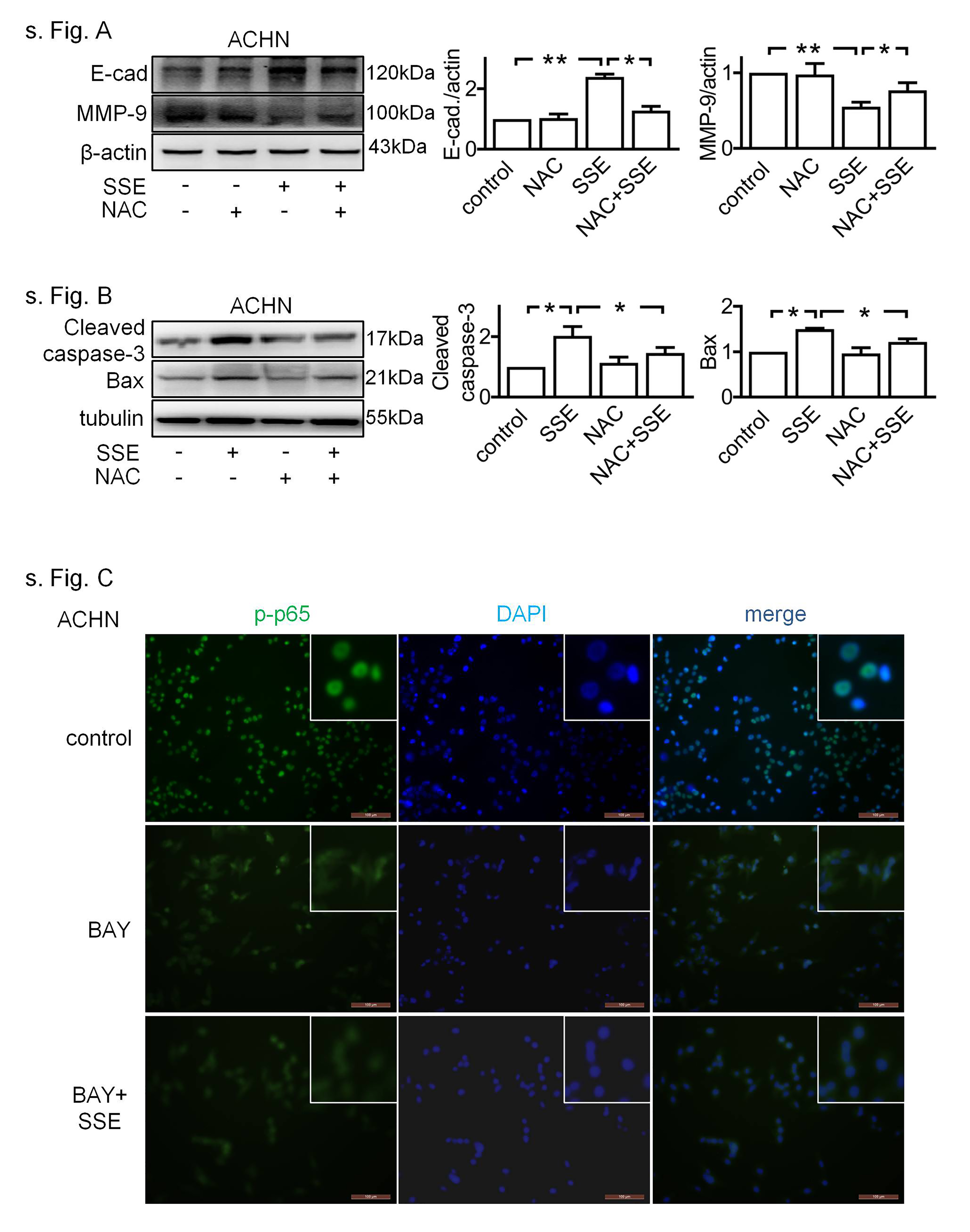

Supplement: Supplementary file 1 — Additional file 1. Supplementary Figure (A) and (B) The ACHN cells were treated with 10 μM SSE, 10 μM NAC, or 10 μM SSE and 10 μM NAC for 6 h. β-Actin or β-tubulin was used as loading controls. The blots shown are representatives. (C) The ACHN cells were treated with 10 μM BAY, or 10 μM BAY and 10 μM SSE for 6 h, the p65 subunit translocation from the cytoplasm to the nucleus was evaluated by immunofluorescence, green spots represent p-p65 staining and blue spots represent the cell nuclei. The images shown are representatives (Scale bar = 100 μm). Data are presented as means ± SD. *P <0.05 and **P < 0.01. [file 12885_2022_9965_MOESM1_ESM.jpg]
